# Association between sitagliptin plus vitamin D3 (VIDPP-4i) use and clinical remission in patients with new-onset type 1 diabetes: a retrospective case-control study

**DOI:** 10.20945/2359-3997000000652

**Published:** 2023-05-29

**Authors:** Marcelo Maia Pinheiro, Felipe Moura Maia Pinheiro, Marcelo Müller de Arruda, Geane Moron Beato, Graciele Alves Corrêa Lima Verde, Georgiana Bianchini, Pedro Rosário Moraes Casalenuovo, Aline Aparecida Agostini Argolo, Lucilene Telles de Souza, Flávia Gomes Pessoa, Thiago Santos Hirose, Eduardo Filgueiras Senra, Camillo Ricordi, Andrea Fabbri, Marco Infante, Susana Nogueira Diniz

**Affiliations:** 1 Univag Centro Universitário Várzea Grande MT Brasil Univag Centro Universitário, Várzea Grande, MT, Brasil; 2 Universidade Anhanguera São Paulo SP Brasil Universidade Anhanguera, São Paulo, SP, Brasil; 3 Beta Cell Center Diabetes & Endocrinologia Cuiabá MT Brasil Beta Cell Center Diabetes & Endocrinologia, Cuiabá, MT, Brasil; 4 Universidade de São Paulo Faculdade de Medicina de São Paulo Hospital das Clínicas São Paulo SP Brasil Hospital das Clínicas, Faculdade de Medicina de São Paulo, Universidade de São Paulo, São Paulo, SP, Brasil; 5 Hospital Universitário Júlio Müller Cuiabá MT Brasil Hospital Universitário Júlio Müller, Cuiabá, MT, Brasil; 6 Universidade de Cuiabá Cuiabá MT Brasil Universidade de Cuiabá (Unic), Cuiabá, MT, Brasil; 7 Clínica Hortênsia Sinop MT Brasil Clínica Hortênsia, Sinop, MT, Brasil; 8 Universidade Federal de São Paulo Escola Paulista de Medicina São Paulo SP Brasil Escola Paulista de Medicina, Universidade Federal de São Paulo (Unifesp), São Paulo, SP, Brasil; 9 Clínica Instituto da Visão Tangará da Serra MT Brasil Clínica Instituto da Visão, Tangará da Serra, MT, Brasil; 10 Medclin Rondonópolis MT Brasil Medclin, Rondonópolis, MT, Brasil; 11 Suprema Clínica e Diagnóstico Rondonópolis MT Brasil Suprema Clínica e Diagnóstico, Rondonópolis, MT, Brasil; 12 Centro Universitário Estácio de Ribeirão Preto Ribeirão Preto SP Brasil Centro Universitário Estácio de Ribeirão Preto, Ribeirão Preto, SP, Brasil; 13 Endocrinoclinica Joinville SC Brasil Endocrinoclinica, Joinville, SC, Brasil; 14 University of Miami Miller School of Medicine Diabetes Research Institute Cell Transplant Center Miami FL USA Diabetes Research Institute (DRI) and Cell Transplant Center, University of Miami Miller School of Medicine, Miami, FL, USA; 15 University of Rome Tor Vergata Diabetes Research Institute Federation Department of Systems Medicine Rome Italy Diabetes Research Institute Federation (DRIF), Department of Systems Medicine, University of Rome Tor Vergata, Rome, Italy; 16 Universal Scientific Education and Research Network Network of Immunity in Infection, Malignancy and Autoimmunity Rome Italy Network of Immunity in Infection, Malignancy and Autoimmunity (NIIMA), Universal Scientific Education and Research Network (USERN), Rome, Italy; 17 Saint Camillus International University of Health Sciences UniCamillus Rome Italy UniCamillus, Saint Camillus International University of Health Sciences, Rome, Italy

**Keywords:** Cholecalciferol, DPP-4 inhibitor, honeymoon phase, type 1 diabetes, vitamin D

## Abstract

**Objective:**

The occurrence of partial remission (honeymoon phase) in type 1 diabetes (T1D) has been associated with a reduced risk of chronic microvascular complications of diabetes. We have published case reports showing that a combination therapy with the DPP-4 inhibitor sitagliptin plus vitamin D3 (VIDPP-4i) can prolong the honeymoon phase in patients with new-onset T1D. In the present case-control study, we investigated the frequency of occurrence of clinical remission (CR) in patients with new-onset T1D after VIDPP-4i treatment.

**Subjects and methods:**

In this case-control study, we collected data spanning 10 years from medical records of 46 patients (23 females) recently diagnosed with T1D. Overall, 27 participants with CR (insulin dose-adjusted glycated hemoglobin [IDAA1c] ≤ 9) at 12 or 24 months composed the case group, and 19 participants without CR served as the control group. Chi-square with Yates correction was used to analyze the association between VIDPP-4i use and CR, and odds ratio (OR) was used to determine the chance of CR due to VIDPP-4i treatment exposure.

**Results:**

In all, 37 patients (80.4%) experienced CR at some time over 24 months. The mean CR duration was 13.15 ± 9.91 months. Treatment with VIDPP-4i was significantly associated with CR. At 24 months, the OR of CR after VIDPP-4i exposure was 9.0 (95% confidence interval [CI] 2.21-30.18, p = 0.0036). Additionally, 9 (33.6%) and 4 (14.8%) patients in the VIDPP-4i group experienced insulin-free CR at 12 and 24 months, respectively.

**Conclusion:**

Therapy with VIDPP-4i was associated with a higher frequency and duration of the honeymoon phase. Randomized controlled trials are needed to confirm these findings.

## INTRODUCTION

Type 1 diabetes (T1D) is an organ-specific autoimmune disease characterized by immune-mediated destruction of insulin-secreting pancreatic beta cells, ultimately resulting in lifelong dependence on exogenous insulin (1). The incidence of T1D peaks between the ages of 5 and 9 years, whereas a second peak occurs at or near puberty (2,3). The main presenting symptoms of new-onset T1D include polyuria, polydipsia, fatigue, and weight loss, which can precede the development of mild-to-moderate or life-threatening diabetic ketoacidosis (DKA) (4,5). Shortly after the onset of clinical T1D and the initiation of insulin therapy, about two-thirds of the individuals with T1D exhibit a transient and partial spontaneous clinical remission phase (also referred to as the “honeymoon phase”), which is accompanied by a substantial reduction in exogenous insulin requirements and near-normal glucose control (6–9). However, population-based cohort studies have shown that approximately 2%-12% of young patients with T1D experience a transient complete remission phase characterized by near-normal glucose control without the need for insulin therapy (7). Significant variability in the overall duration of the remission phase exists among patients with T1D, with an average duration of approximately 7 months (10). It has been suggested that both metabolic and immune factors may contribute to beta-cell recovery during the remission phase. These factors include a transient recovery of immune tolerance to beta-cell autoantigens (which may involve T-cell regulatory pathways), as well as improvement in insulin sensitivity and decreased glucotoxicity secondary to the improvement in glucose homeostasis achieved after the initiation of insulin therapy (6,11).

The most widely accepted definition of partial remission of T1D is based on calculated insulin dose-adjusted glycated hemoglobin A1c (IDAA1c) ≤ 9, which corresponds to a predicted stimulated C-peptide value > 300 pmol/L (>0.9 ng/mL) (12). The IDAA1c is a more reliable marker of partial remission compared with glycated hemoglobin A1c (HbA1c) alone or insulin dose ≤ 0.5 units · kg^−1^ · 24 h^−1^, which misclassifies a subset of patients early in the disease course because of a lack of or delay in insulin therapy around the time of diagnosis (12). Occurrence of partial remission has been associated with a reduced risk of chronic microvascular complications of diabetes (13). Accordingly, retention of endogenous insulin secretion after T1D diagnosis has been associated with better glucose control, lower risk of hypoglycemia, lower insulin requirements, and reduced incidence of retinopathy and nephropathy (14–17). Then, recognizing the clinical, laboratory, and therapeutic characteristics involved in the occurrence and maintenance of the honeymoon phase are important for understanding this phenomenon and preventing diabetes complications.

In 2016, we published the first report of a markedly prolonged clinical remission phase (up to 4 years) and preserved beta-cell function in two patients with T1D who received the dipeptidyl peptidase-4 inhibitor (DPP-4i) sitagliptin (100 mg/day) plus vitamin D3 (5,000 IU/day) (18). After 10 years, these patients remain in remission, and one of them (included in the present study) is currently insulin-free. We also recently published a review article discussing the rationale for investigating the combination therapy with DPP-4 inhibitors (DPP-4is) and vitamin D (also referred to as “VIDPP-4i”) as a potential immunomodulation strategy aimed to prevent disease progression and preserve beta-cell function in patients with autoimmune diabetes (19). Of note, we proposed that vitamin D and DPP-4i may exert synergistic antiinflammatory and immunomodulatory properties when administered together in patients with new-onset autoimmune diabetes. We conducted the present retrospective case-control study to investigate the association between VIDPP-4i use and T1D clinical remission.

## SUBJECTS AND METHODS

### Study design and participants

The data of this case-control study were retrospectively collected from medical records of patients seen at outpatient clinics in the participating institutions from May 25, 2012 (date of the first prescription of VIDPP-4i) to May 31, 2022. A total of 46 patients with newly diagnosed T1D were analyzed in this study. The diagnosis of T1D was established according to the American Diabetes Association (ADA) criteria, including HbA1c ≥ 6.5% and/or fasting plasma glucose ≥ 126 mg/dL, exogenous insulin requirement from diagnosis, and positivity for at least one of the T1D-associated autoantibodies (islet autoantibodies) including insulin autoantibodies (IAAs; radioimmunoassay normal value < 7.3% binding), glutamic acid decarboxylase autoantibodies (GADA; chemiluminescence, normal value < 17 IU/mL), islet antigen-2 antibodies (IA-2A; chemiluminescence, normal value < 28 IU/mL), islet cell autoantibodies (ICA; indirect immunofluorescence, normal value is “nonreactive”), or zinc transporter 8 autoantibodies (ZnT8A; enzyme immunoassay, normal value <15 UA/mL) (20).

**Figure 1:**
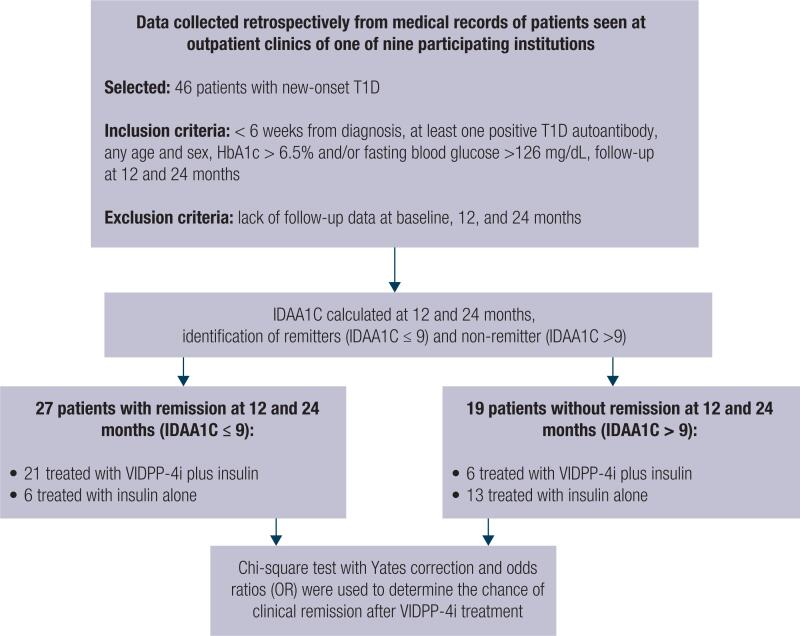
Flowchart of our retrospective study design.

A total of 27 participants with clinical remission at 12 or 24 months served as the case group, and 19 participants without clinical remission at 12 or 24 months served as the control group. Patients exhibiting an IDAA1c value ≤ 9 at 12 or 24 months after T1D diagnosis were defined as remitters. On the other hand, non-remitters exhibited an IDAA1c value > 9 at 12 and 24 months (Figure 1). Both groups were matched for sex and age.

### Data collection, informed consent, and ethical approval

Demographic, anthropometric, clinical, and laboratory data were retrospectively collected through a medical record review that was independently performed by two members of our research team. Data were collected via electronic medical records and recorded in an anonymous outpatient T1D database containing unambiguous and alphanumeric codes that were progressively assigned to all study participants. Overall, all participants underwent a follow-up medical visit every 3-4 months. The Research Ethics Committee of *Universidade Anhanguera de São Paulo* (Unian – SP) approved the collection of data from medical records for publication (registration number 4.657.744/2021), as this was a retrospective case-control study. The CAAE NUMBER (Certificate of Presentation for Ethical Appreciation) generated at Plataforma Brasil is 43875421.5.0000.5493.

### Clinical and biochemical evaluation

Fasting plasma glucose and HbA1c were measured as markers of glucose homeostasis, whereas fasting plasma C-peptide level was measured as a marker of residual beta-cell function at diagnosis. Total serum 25-hydroxyvitamin D (25(OH)D) levels – including 25(OH)D2 and 25(OH)D3 – were also measured, as 25(OH)D represents the most reliable biomarker of vitamin D status (21,22). Total serum 25(OH)D levels were measured using chemiluminescent immunoassay. Vitamin D sufficiency was defined by serum 25(OH)D concentration ≥ 20 ng/mL. Fasting C peptide (normal value 1.10-4.4 ng/mL – chemiluminescence), glycemia (normal value 70-99 mg/dL – hexokinase method), HbA1c (normal value < 5.7% – high-performance liquid chromatography), and total serum 25(OH)D were measured in the morning after an 8-hour fast.

Anthropometric measurements (height, body weight) and demographic parameters (age, sex) were collected. Linear height (cm) was measured using a Harpenden Stadiometer. Body mass index (BMI) was calculated by dividing the body weight in kilograms by the square of the height in meters (kg/m^2^). We also calculated the BMI z-scores as measures of relative weight adjusted for child age and sex (<20 years). The BMI z-scores were calculated using a pediatric BMI z-score calculator (Pediatric Z-Score Calculator for patients between 2 and 20 years of age, Children's Hospital of Philadelphia Research Institute; available at: https://zscore.research.chop.edu/. Accessed: September 25, 2022). Ethnic definition was not considered in this retrospective analysis due to the high degree of miscegenation of the Brazilian population and the absence of this information in some medical records. We also collected relevant clinical data, including the occurrence or not of DKA at diagnosis, as well as data on total daily insulin dose, expressed as IU/kg of body weight/day), when available, and number of patients who were insulin-free at different time points during the follow-up period (12-24 months). We assessed the occurrence of partial clinical remission of T1D by calculating the IDAA1c using the following formula: HbA1c (%) + [4 × insulin dose (units per kg per 24 hours)]. An IDAA1c value ≤ 9 within 1 month from T1D diagnosis was considered indicative of partial clinical remission, as previously reported (12). Insulin-free clinical remission was defined as the withdrawal of insulin therapy, which was carried out when a patient experienced recurrent episodes of fasting and/or postprandial hypoglycemia even with low insulin doses (<0.3 IU/kg of body weight/day) and with HbA1c values < 6.5%.

### Statistical analysis

The association between sitagliptin plus vitamin D3 (VIDPP-4i) use and clinical remission (honeymoon phase) was analyzed using chi-square with Yates correction. Odds ratio (OR) was used to determine the chance of clinical remission due to VIDPP-4i treatment exposure. The Mann-Whitney test was used as a nonparametric test. Data are shown as mean ± standard deviation (SD). A p value of less than 0.05 was considered statistically significant. Statistical analysis and graph generation were performed using GraphPad Prism Software, version 9.2.0 (GraphPad Software, San Diego, CA, USA).

## Results

This retrospective case-control study included 46 consecutive patients with newly diagnosed T1D seen at one of the nine participating outpatient diabetes centers in Brazil. Baseline data are shown in Table 1. When available, data at each visit were used for statistical analysis up to 24 months. In all, 27 patients were treated with insulin plus VIDPP-4i, and 19 patients were treated with insulin alone. All patients received dietary management and standard intensive treatment of a basal-bolus insulin regimen. Specifically, 36 patients were using glargine insulin, 6 were using degludec insulin, and 4 were using NPH insulin as basal insulin. As bolus insulin, 36 were using rapid-acting insulin (lispro, aspart, or glulisine) and 4 were using regular insulin. There was no difference in total daily insulin dose between groups. All patients checked their capillary blood glucose levels at home. Of note, 23 patients older than 7 years were treated with sitagliptin 100 mg/day plus vitamin D3 5,000 IU/day, whereas 4 patients younger than 7 years were initially treated with sitagliptin 50 mg/day (empirical dose adjustment employed by their doctors) and vitamin D3 2,000 IU/day. Vitamin D3 doses were progressively adjusted to achieve and maintain serum 25(OH)D levels between 40-60 ng/mL. Conversely, sitagliptin dose remained unchanged throughout the follow-up period (12-24 months). All patients started VIDPP-4i treatment within 6 weeks after T1D diagnosis. Only one 5-year-old male participant, who did not experience clinical remission at 12 months, interrupted sitagliptin use, as per his family's decision. The mean duration of partial remission was 13.15 ± 9.91 months (95% confidence interval [CI] 10.24-16.06, range 0-24 months). A total of 37 (80.4%) patients experienced clinical remission at any time (defined by an IDAA1c value ≤ 9). When comparing remitters *versus* non-remitters at 12 and 24 months, only BMI at baseline and VIDPP-4i use showed statistical difference (Table 2). However, when the BMI was adjusted for z-score, the statistical difference disappeared. Therefore, only VIDPP-4i exposure seems to have influenced the occurrence of clinical remission in our cohort. At 12 months, the OR of clinical remission after VIDPP-4i exposure was 4.28 (95% CI 1.127-15.32, p = 0.064). At 24 months, the OR of clinical remission after VIDPP-4i exposure was 9.0 (95% CI 2.21-30.18, p = 0.0036). Of note, 9 (33.6%) and 4 (14.8%) patients in the VIDPP-4i exposure group experienced insulin-free clinical remission at 12 months (mean HbA1c 5.68 ± 0.59%, 95% CI 5.23-6.14%) and 24 months (mean HbA1c 5.7 ± 0.46%, 95% CI 4.95-6.44%), respectively. On the contrary, insulin-free clinical remission did not occur in any patient treated with insulin alone.

**Table 1:** Demographic, anthropometric, clinical, and laboratory characteristics of the participants at the time of diagnosis (baseline)

	(n = 46)
Age at diagnosis (years)	12.72 ± 6.44 (4-37)
Females (n, %)	23 (50%)
Body weight (kg)	42.11 ± 16.58
Height (m)	1.50 ± 0.20
BMI (kg/m^2^)	18.96 ± 3.53
BMI z-score	-0.60(±1.45)
DKA at diagnosis (n, %)	19 (41.3%)
FPG (mg/dL)	275.5 ± 117.9
HbA1c (%)	10.67 ± 2.70
TDD (IU/kg of body weight/24h)	0.49 ± 0.27
25(OH)D (ng/mL)	32.41 ± 13.87
Fasting C peptide (ng/mL)	0.82 ± 0.53
GADA antibody (IU/mL) (n = 46)	37(80.4%)*
Islet antigen-2 antibody (IA-2A) (IU/mL) (n = 3)	2*(66.6%)*
Insulin autoantibody (<7.3% binding) (n = 14)	5*(35.7%)*
Islet cell autoantibody (ICA - nonreactive) (n = 18)	10*(55.5%)*
Zinc transporter 8 autoantibody (ZnT8A – UA/mL) (n = 2)	2*(100%)*

Data are presented as mean ± standard deviation or frequency (percentage). Abbreviations: 25(OH)D, serum 25-hydroxyvitamin D levels; BMI, body mass index; DKA, diabetic ketoacidosis; FPG, fasting plasma glucose; GADA antibody, glutamic acid decarboxylase autoantibody; HbA1c, glycated hemoglobin; n, number of patients tested for the antibody; TDD, total daily insulin dose.

* Number and % of patients tested who were positive for the antibody.

**Table 2:** Baseline demographic data of the participants and comparison of anthropometric, clinical, and laboratory characteristics and use of combination therapy with DPP-4 inhibitor plus vitamin D between remitters and non-remitters

	Remitters (n = 27)	Non-remitters (n = 19)	P value
Age at diagnosis (years)	13.89 ± 7.0	11.05 ± 5.1	0.056
Females – n (%)	12 (44.4%)	11 (57.8%)	0.54
Males – n (%)	15 (55.5%)	8 (42.1%)	0.54
BMI (kg/m^2^)	19.07 ± 3.68	15.86 ± 2.49	**0.002**
BMI z-score	-0.26 ± 1.12	-1.12 ± 1.69	0.11
DKA at diagnosis – n (%)	10 (38.46%)	9 (47.37%)	0.76
FPG (mg/dL)	262.4 ±101.9	294.1 ± 138.4	0.43
HbA1c (%)	10.7 ± 2.60	10.5 ± 2.91	0.78
TDD (IU/kg of body weight/24h)	0.46 ± 0.31	0.53 ± 0.21	0.21
25(OH)D (ng/mL)	29.55 ± 14.9	36.78 ± 9.29	0.066
Fasting C peptide (ng/mL)	0.81 ± 0.49	0.84 ± 0.69	0.060
Hashimoto's thyroiditis	5 (18.5%)	3(15.78%)	0.715
VIDPP-4i use	21 (77.78%)	6 (31.58%)	**0.0025**

Data are presented as mean ± standard deviation or frequency (percentage). Abbreviations: 25(OH)D, serum 25-hydroxyvitamin D levels; BMI, body mass index; DKA, diabetic ketoacidosis, FPG, fasting plasma glucose; HbA1c, glycated hemoglobin; n, number of patients with data available for statistical analysis; SD, standard deviation; TDD, total daily insulin dose; VIDPP-4i, combination therapy with DPP-4 inhibitor plus vitamin D.

The mean HbA1c values in remitters and non-remitters were, respectively, 6.0 ± 0.69% and 8.42 ± 2.5% (p < 0.0001) at 12 months and 6.21 ± 0.62% and 9.28 ± 2.22% (p < 0.0001) at 24 months.

Regarding fasting C peptide, the mean levels in remitters and non-remitters, respectively, were 0.95 ± 0.44 ng/mL and 0.42 ± 0.25 ng/mL (p = 0.0223) at 12 months and 0.73 ± 0.42 ng/mL and 0.25 ± 0.24 ng/mL (p = 0.0112) at 24 months.

Unfortunately, most patients had only one or two T1D-related autoantibodies tested, preventing an adequate statistical analysis (Table 1).

Regarding vitamin D status, the mean serum 25(OH)D levels at baseline (32.41 ± 13.87 ng/mL) indicated vitamin D sufficiency (≥20 ng/mL). Notably, the mean serum 25(OH)D level was < 20 ng/mL in 5 patients (16.1%) and >30 ng/mL in 18 patients (58.1%). At 12 and 24 months, there was no difference in vitamin D levels between remitters and non-remitters. However, at 24 months, patients in the VIDPP-4i group showed significantly higher levels of vitamin D than controls (53.51 ± 16.54 ng/mL *versus* 33.63 ± 6.07 ng/mL, respectively, p = 0.0007).

The only other autoimmune disease identified was Hashimoto's thyroiditis, which was diagnosed in 8 patients (Table 2).

From our retrospective chart review, we did not detect any severe adverse drug event (ADE) related to the use of VIDPP-4i, including alterations in complete blood count, alterations in markers of liver and kidney function, respiratory tract infections or other systemic infections, bullous pemphigoid and other skin disorders, gastrointestinal symptoms, hypercalcemia, hypercalciuria, nephrolithiasis, or severe hypoglycemia.

## Discussion

Our retrospective case-control study suggests that VIDPP-4i (*i.e.*, vitamin D3 plus sitagliptin) exposure in addition to insulin therapy may substantially prolong the duration of the clinical remission phase in a population of children, adolescents, and young adults with new-onset T1D. Specifically, patients in this study exhibited a longer duration of the partial remission phase compared with those in the main studies available in the literature (Table 3). Furthermore, approximately one-third and one-seventh of the patients in the VIDPP-4i exposure group experienced insulin-free clinical remission at 12 and 24 months, respectively. This finding is particularly important due to the rare occurrence of insulin-free clinical remission (complete remission) during the natural history of T1D (7,23,24).

**Table 3:** Main studies analyzing the prevalence of clinical remission in children and adolescents with new-onset type 1 diabetes

Study (Country)	n	Age (years)	(F/M) %	DKA %	PR %	DR (Months)	% PR 12 Months	%PR 24 Months	%IFCR 12 Months
Present study, Brazil	46	12.72	50/50	41.3	80.4	13.15	62	51.2	19.5
Camilo and cols., 2020, Brazil	51	10.1	50.9/49.1	29.3	41.2	7	33.3	7.84	0
Bektas and cols., 2020, Turkey	55	10.6	43.7/56.3	47	56.4	NR	21.8	5.5	0
Pecheur and cols., 2014, Belgium	242	8.8	47.5/52.5	25.6	56.2	9.2	28.4	7.5	0
Chiavaroli and cols., 2019, New Zealand	678	9.2	46.3/53.7	27.1	42.4	NR	10.6	7.1*	0
Nagl and cols., 2017, Germany and Austria	3657	7.9	49/51	15	61	9	28	13	0

Abbreviations: n: number of patients, age: mean or median age of the participants at each study, F: female; M, male; DKA: diabetic ketoacidosis; NR: not reported; PR: partial clinical remission at any time; DR: duration of remission; IFCR: insulin-free clinical remission. All studies used IDAA1c [HbA1c (%) + [4 × insulin dose (units per kilogram per 24 h)] to define the occurrence of partial clinical remission. An IDAA1c value ≤9 was considered indicative of partial clinical remission.

* Data assessed at 18 months.

Whether the possible prolongation of the remission phase observed was based on metabolic or immunomodulatory effects (or a combination of both) exerted by VIDPP-4i remains uncertain. In this regard, we recently published a review of the current evidence on the potential role of VIDPP-4i as an immunomodulation therapy aimed to counteract beta-cell autoimmunity and preserve beta-cell mass and function in autoimmune diabetes (19). Over the last years, preclinical data and preliminary clinical studies have suggested that vitamin D and DPP-4i may exert synergistic antiinflammatory and immunomodulatory effects by acting on the vitamin D receptor and DPP-4 enzyme expressed by different types of immune cells. Of note, vitamin D and DPP-4is appear to upregulate regulatory T cells (Tregs) and affect the polarization of T-helper cells (CD4+ T cells or Th cells) by increasing Th2 cells and inhibiting Th1 and Th17 cell development, thereby promoting a shift of T lymphocytes from an “effector” toward a “regulatory” phenotype (19,25). Also, vitamin D and DPP-4i can reduce the differentiation and activation of autoreactive CD8+ (cytotoxic) T cells and B cells, resulting in reduced islet autoantibody production and decreased islet inflammation (insulitis) and beta-cell apoptosis (19,26). Moreover, the VIDPP-4i combination therapy has been shown to provide beneficial antiinflammatory effects in different chronic diseases such as type 2 diabetes (27,28), diabetic nephropathy (29), and mononeuritis multiplex (30,31).

In line with this hypothesis, our previous preliminary data obtained from a smaller cohort of patients (n = 46; 26 patients with T1D and 20 controls) showed that a VIDPP-4i regimen (with sitagliptin 100 mg/day plus vitamin D3 5,000 IU/day) in addition to insulin therapy was associated with a prolonged clinical remission phase (mean duration of 27.1 ± 18.9 months), along with a significant decrease in CD8+CD26+ T-cell count compared with a regimen with insulin alone in patients with T1D (32,33). Similar findings regarding the efficacy of VIDPP-4i in preserving residual beta-cell function have been confirmed in case reports (34) and randomized controlled trials (35) involving patients with latent autoimmune diabetes in adults (LADA), a distinct form of autoimmune diabetes characterized by a less severe immune-mediated beta-cell destruction, an older age of onset, and a slower progression toward insulin dependence compared with T1D. Importantly, in such studies, VIDPP-4i was safe and well tolerated, with no side effects observed throughout the follow-up period (33–35).

The literature regarding variables and markers associated with higher or lower rates of the remission phase in patients with T1D has yielded diverging results. Higher basal and stimulated C-peptide levels (36,37), younger age and higher BMI at diagnosis (38), higher insulin sensitivity (39), male sex (40–42), lower initial HbA1c level at diagnosis (40,42,43), age at T1D onset ≥ 5 years (42–45), initial islet autoantibody negativity (42), and disease onset without DKA (42) have been associated with a higher likelihood of experiencing the remission phase across different studies conducted on patients with T1D. In our case-control study, the mean BMI and VIDPP-4i exposure were the exclusive parameters that differed significantly between remitters and non-remitters at baseline, with higher BMI and VIDPP-4i exposure observed in remitter subjects. However, when we adjusted the BMI by z-score, the statistical difference in BMI disappeared. With regard to the age of the study participants at the time of T1D diagnosis, younger age at T1D diagnosis has been associated with lower residual beta-cell function (24,46), more pronounced decline in stimulated C peptide (43,45), and lower occurrence of the partial clinical remission phase (6,10). Furthermore, Leete and cols. (47) recently showed statistically significant differences in stimulated C-peptide levels and proinsulin-to-C-peptide ratio values between patients diagnosed with T1D before the age of 7 years and patients diagnosed with T1D at the age of ≥ 13 years. In contrast, age does not seem to have affected the results of our study.

The rates of partial remission achieved in our study at 12 and 24 months (62% and 51.2%, respectively) (Table 3) and the mean duration of the partial remission (13.15 months) (Table 3) were markedly greater than the corresponding results observed in other studies (23,36,37,43,44). In a recent Brazilian study involving 51 children and adolescents with new-onset T1D, Camilo and cols. (46) showed that partial remission occurred in 41.2% of the patients until 3 months after the T1D diagnosis. The median duration of partial remission was 7 months. After a median follow-up of 13 months, 81% of the patients ended the remission phase, while only 19% remained in partial remission phase. At diagnosis, remitters had significantly higher median C-peptide levels than non-remitters (0.80 ng/mL *versus* 0.30 ng/mL, respectively), whereas no other differences in autoantibody profile or clinical and metabolic manifestations were seen between remitters and non-remitters (46). In our study, the baseline fasting C-peptide level did not affect the results. These data further support the understanding that the results observed in our retrospective study in terms of rates of insulin-free clinical remission, partial remission, and duration of the remission phase are likely due to the VIDPP-4i exposure rather than other demographic, anthropometric, or clinical factors. Of note, the mean HbA1c values at 12 and 24 months in remitters remained < 6.5%, and these values were lower than those observed in other studies (9,10,24). Furthermore, the statistically significant difference in C-peptide levels at 12 and 24 months between remitters and non-remitters appears to be related to the use of VIDPP-4i, as these levels remained stable during the 24-month follow-up period.

Importantly, our retrospective chart review did not reveal any severe ADE related to the use of VIDPP-4i, suggesting that this combination therapy was safe and well tolerated. Although DPP-4is are not approved for use in the pediatric population, a recent study conducted in youths (aged 10-17 years) with type 2 diabetes found that sitagliptin was generally well tolerated and had a safety profile similar to that reported in adults (48). Safety of DPP-4is for the treatment of type 2 diabetes in children and adolescents was also confirmed in a recent meta-analysis (49).

Based on these data, although DPP-4is are not approved for the treatment of T1D, we recommend the investigation of these drugs (in combination with vitamin D) as a potential immunomodulation therapy for patients with autoimmune diabetes. Indeed, evidence suggests that sitagliptin and other DPP-4is are able to reduce daily insulin requirements and improve glucose control without increasing the risk of hypoglycemia or compromising the glucagon counterregulatory response during hypoglycemia in patients with T1D (19,50–52).

We acknowledge that our study has various limitations, including a small number of patients, the retrospective design, and the missing data regarding some parameters (like HbA1c and C peptide at some time points). In addition, we could not define the severity of DKA at diagnosis as the medical records only reported its presence or absence. However, in a recent publication, Clapin and cols. (53) documented that only the presence of moderate-to-severe DKA (but not mild DKA) at T1D diagnosis may affect long-term metabolic control, as evidenced by HbA1c values. Furthermore, some studies demonstrated the importance of the number of islet autoantibodies in T1D remission rates (54–57), but we were unable to analyze the influence of T1D-related autoantibodies on the results of our study, as most patients had only one or two autoantibodies measured.

Regarding insulin therapy, nearly 90% of our patients were on a basal-bolus regimen with insulin analogues. Some studies have shown no differences in partial remission rates and residual beta-cell function in T1D when comparing continuous subcutaneous insulin infusion *versus* multiple daily injections (58–60). Therefore, the insulin regimen may not have influenced our results.

Although this study was a retrospective case-control analysis, its main strength is represented by the fact that it is the first publication evaluating the effects of VIDPP-4i exposure on the honeymoon phase in a group of patients with new-onset T1D. We emphasize that IDAA1c, used in our study, is the gold standard for the definition of partial remission (61–63). Furthermore, the statistical significance appeared only at 24 months after VIDPP-4i exposure, supporting the hypothesis that the prolongation of the clinical remission phase may be due to this treatment, since clinical remission phases longer than 12 months are infrequent (63,64).

In conclusion, we have shown that VIDPP4-i exposure (in addition to insulin therapy) may increase the rates and duration of the honeymoon phase in patients with new-onset T1D. Additionally, VIDPP-4i was safe and well tolerated, even in children younger than 7 years. Future prospective studies are needed to confirm our preliminary findings, as well as all the aforementioned hypotheses.
